# A Novel Phage-Library-Selected Peptide Inhibits Human TNF-α Binding to Its Receptors

**DOI:** 10.3390/molecules19067255

**Published:** 2014-06-03

**Authors:** Jlenia Brunetti, Barbara Lelli, Silvia Scali, Chiara Falciani, Luisa Bracci, Alessandro Pini

**Affiliations:** 1 Department of Medical Biotechnologies, University of Siena, Via A. Moro 2, 53100 Siena, Italy; E-Mails: jlenia.brunetti@unisi.it (J.B.); barbara.lelli@unisi.it (B.L.); silvia.scali@unisi.it (S.S.); luisa.bracci@unisi.it (L.B.); 2 SetLance srl, via Fiorentina 1, 53100 Siena, Italy; E-Mail: falciani@setlance.com; 3 Laboratorio di Patologia Clinica, Azienda Ospedaliera Universitaria Senese, 53100 Siena, Italy

**Keywords:** TNF-α, anti-TNF-α peptide, branched peptides, phage display, solid-phase synthesis, competitive selection, surface plasmon resonance

## Abstract

We report the identification of a new human tumor necrosis factor-alpha (TNF-α) specific peptide selected by competitive panning of a phage library. Competitive elution of phages was obtained using the monoclonal antibody adalimumab, which neutralizes pro-inflammatory processes caused by over-production of TNF-α *in vivo*, and is used to treat severe symptoms of rheumatoid arthritis. The selected peptide was synthesized in monomeric and branched form and analyzed for binding to TNF-α and competition with adalimumab and TNF-α receptors. Results of competition with TNF-α receptors in surface plasmon resonance and melanoma cells expressing both TNF receptors make the peptide a candidate compound for the development of a novel anti-TNF-α drug.

## 1. Introduction

Tumor necrosis factor-alpha (TNF-α) is a potent pro-inflammatory cytokine exerting pleiotropic effects on various cell types. TNF-α elicits a wide variety of responses, including fever, synthesis of acute-phase proteins, increased vascular permeability, T and B-cell activation and migration, cell proliferation and apoptosis [1,2,3]. TNF is translated as a 26 kDa type II transmembrane protein that assembles into homo-trimers displayed on the cell surface of macrophages, lymphocytes and other cell types [4,5]. Each subunit of the membrane trimer is cleaved at the cell surface by the metalloproteinase ADAM17 (also called TNF-α-converting enzyme: TACE), producing a soluble form of TNF-α trimer with a subunit mass of 17 kDa. Two specific cell surface receptors, TNFR1 and TNFR2, mediate the biological actions of TNF-α in either soluble or cell surface forms [6,7,8,9,10,11,12]. In the immune system, TNF-α is involved in the acute inﬂammatory response to stimuli, such as infection or tissue injury, and also plays a critical role in the pathogenesis of chronic inflammation and chronic inﬂammatory diseases, such as rheumatoid arthritis (RA) and Crohn’s disease [13]. TNF-α also plays a central role in the pathogenesis of psoriasis, psoriatic arthritis and ankylosing spondylitis [6]. Given its pivotal role in many inflammatory diseases, TNF-α has been proposed as a therapeutic target for a number of diseases. Five anti-TNF-α agents (infliximab, etanercept, adalimumab, certolizumab pegol and golimumab) have been successfully introduced for the treatment of chronic inflammatory diseases. All anti-TNF-α agents are effective against RA, though with very different efficacies, and not all of them are effective against Crohn’s disease [14]. Thus the need for new anti-TNF drugs is still pressing. Here we report the selection of a novel anti-human TNF-α peptide from a phage library, its synthesis as a tetra-branched molecule and *in vitro* characterization of the branched peptide for inhibition of TNF-α binding to its receptors, which is the first property for an anti-TNF-α agent to be considered a potential drug for anti-inflammatory therapies.

## 2. Results and Discussion

### 2.1. Phage Display and Synthesis

A commercial phage library (Ph.D.12, NEB) was used to identify 12-mer peptides that bind human TNF-α (hTNF-α). Three cycles of biopanning were carried out against soluble biotinylated hTNF-α. Elution of specific anti-TNF-α peptides was achieved by addition of adalimumab monoclonal antibody. A parallel biopanning was carried out by surface plasmon resonance (SPR) using Biacore T100. For this selection the same phage library was injected over biotinylated hTNF-α, previously immobilized on a streptavidin (SA) sensor chip, and bound phages were eluted by competition with adalimumab monoclonal antibody. The phage ELISA performed to detect TNF-α-specific peptides, selected by conventional biopanning or BiacoreT100 phage selection, revealed several positive clones, which DNA analysis showed to express the same amino acid sequence: HIHDDLLRYYGW.

**Figure 1:**
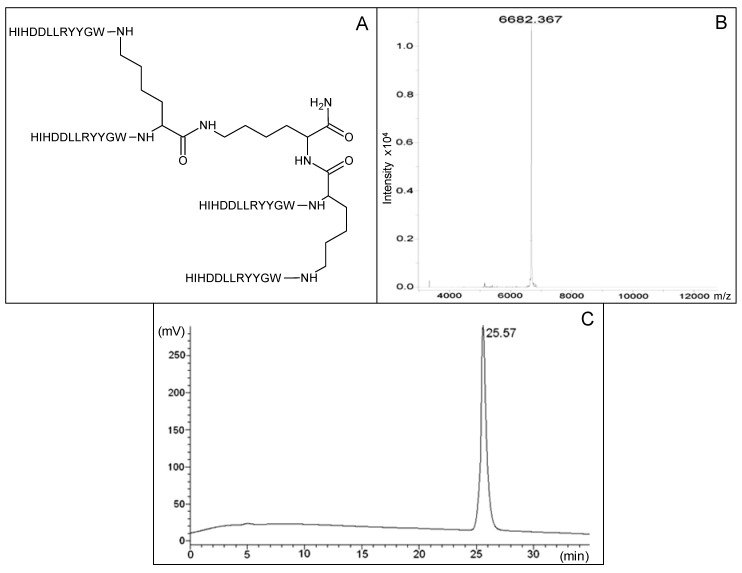
(**A**) Structure of human anti-TNF-α peptide synthesized in tetra-branched form. Amino acids of the four peptide sequences are indicated as one letter code. (**B**) MS profile of purified peptide, analysis of sample was performed with a Bruker Daltonic ultraflex MALDI TOF/TOF mass spectrometer. MW is indicated. (**C**) RP-HPLC profile of human anti-TNF-α peptide after purification. Retention time is indicated.

The selected peptide was synthesized in a tetrameric form (Figure 1A) through standard Fmoc solid phase synthesis, building the tetramer on a three-lysine core [15]. The peptide was purified by HPLC and controlled by MS (Figure 1B,C), as described in the experimental section. The tetra-branched peptide structure was chosen because it strongly increases peptide stability to circulating proteases, thus improving half-life with respect to corresponding monomeric sequences [16,17,18]. The branched structure constructed with short peptides is generally non immunogenic [19]. Stability and low immunogenicity are of great importance for possible use *in vivo* [20,21,22,23,24,25]. In order to allow their immobilization on a Biacore streptavidin-coated chip, both the tetra-branched peptide and the corresponding monomeric sequence were also synthesized with a C-terminal biotin. 

### 2.2. Binding Experiments by SPR

**Figure 2:**
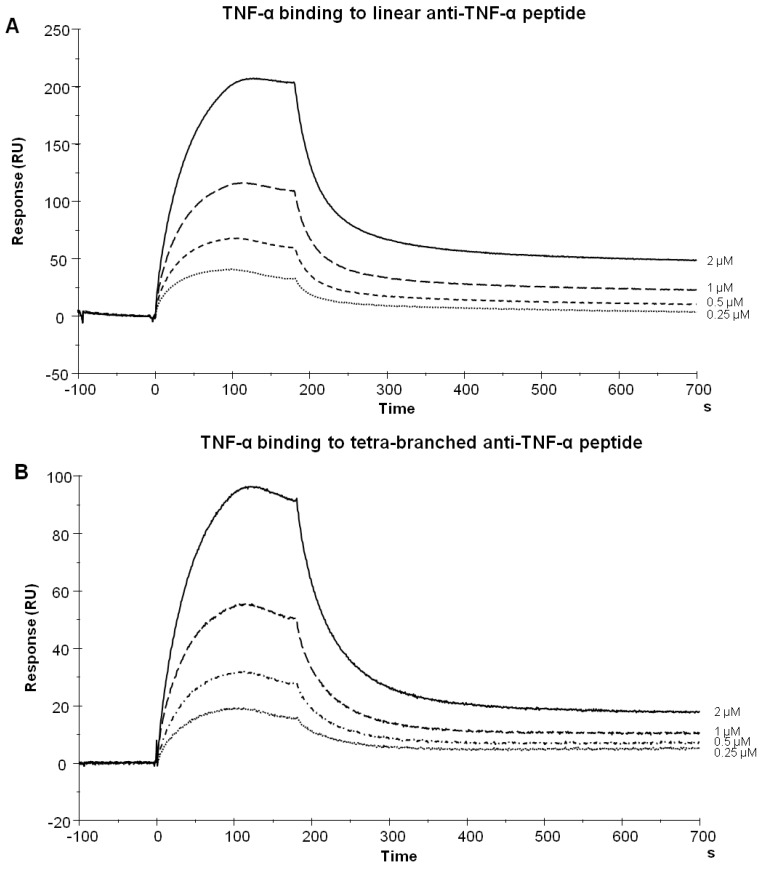
Biacore sensorgrams illustrating hTNF-α binding to the immobilized peptides. TNF-α, from 250 nM to 2 µM, was injected over biotin-coupled linear (A) or tetra-branched (B) peptide immobilized on a SA sensor chip.

hTNF-α was injected over linear and tetra-branched peptides previously immobilized on a Biacore T100 SA sensor chip. hTNF-α was flowed at concentrations ranging from 250 nM to 2 µM. To assess any non-specific binding, hTNF-α was also injected over an empty flow cell. The linear (Figure 2A) and tetra-branched peptides (Figure 2B) bound hTNF-α in a dose-dependent manner. The peptides proved specific for hTNF-α since they did not show any binding for murine TNF-α (not shown). The TNF-α-specific peptide synthesized in linear and tetrameric form showed practically the same kinetics (KD 3 × 10^−6^ M and 6 × 10^−6^ M, respectively).

### 2.3. Human TNF-α Binding to Adalimumab was Inhibited by Tetra-Branched Anti-TNF-α Peptide

**Figure 3:**
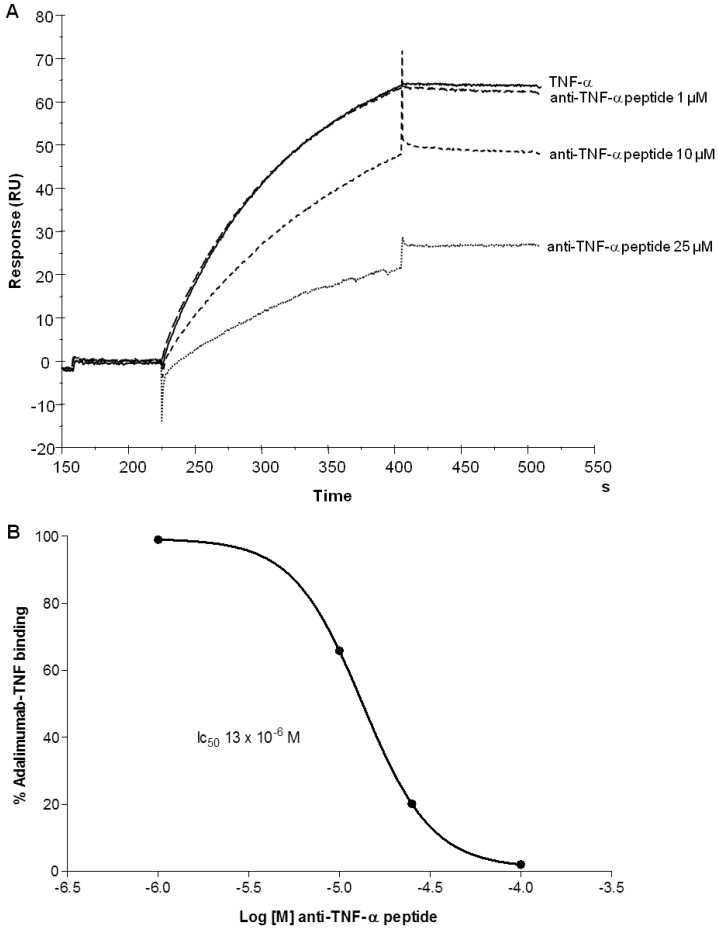
Inhibition of hTNF-α binding to adalimumab by branched anti-TNF-α peptide in SPR. A, adalimumab was captured via Protein A on a CM5 sensor chip as described in the Experimental Section. hTNF-α was flowed in the presence of different concentrations (1, 10 and 25 µM) of branched anti-TNF peptide. B, Curve of binding inhibition of hTNF-α binding to adalimumab derived by experiment in A. y axis indicates the percentage of binding between adalimumab and hTNF-α; x axis represents the concentration of tetra-branched anti-TNF peptide. The IC_50_ is indicated.

The anti-hTNF-α monoclonal antibody adalimumab was captured on a protein-A-coated sensor chip. 10 nM hTNF-α was injected over the immobilized antibody in the presence of different concentrations (25, 10 and 1 µM) of the tetra-branched anti-TNF-α peptide (Figure 3A). 25 µM of tetrabranched anti-TNF-α peptide inhibited about 80% of TNF-α binding to adalimumab with an IC_50_ of 13 × 10^−6^ M (Figure 3B). This test confirmed that the anti-TNF-α peptide and adalimumab bound the target protein at the same site.

### 2.4. Branched Anti-TNF-α Peptide Inhibited Binding of TNF-α to TNF Receptor2

**Figure 4:**
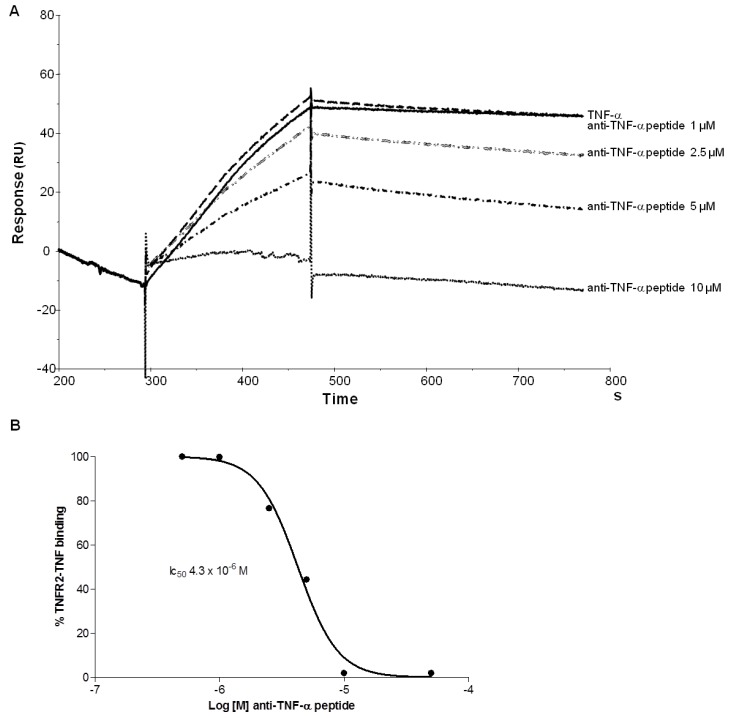
Inhibition of TNF-α binding to TNFR2 analysed by SPR. A, Biacore sensorgrams illustrating binding of 3 nM hTNF-α to immobilized TNFR2, in the presence of different concentrations of branched peptide, from 10 µM to 1 µM. B, Curve of binding inhibition of hTNF-α to TNFR2 derived by experiment in A. y axis indicates the percentage of binding between TNFR2 and TNF-α; x axis represents the concentration of tetra-branched anti-TNF peptide. In B panel peptide concentrations of 500 nM and 50 µM are also reported. The IC_50_ is indicated.

We tested the ability of branched anti-TNF-α peptide to inhibit hTNF-α binding to TNFR 2 by SPR. A chimeric recombinant TNFR2-Fc was captured on a CM5 sensor chip, which had previously been coated with Protein A. hTNF-α was then flowed over TNFR2 in the presence of different concentrations of the branched anti-TNF-α peptide. The anti-TNF-α peptide completely inhibited TNF-α binding to TNF receptor 2 (Figure 4A), with an IC_50_ of 4.3 × 10^−6^ M (Figure 4B).

### 2.5. Branched Anti-TNF-α Peptide Inhibited Binding of TNF-α to Cells Expressing TNF-α Receptors

**Figure 5:**
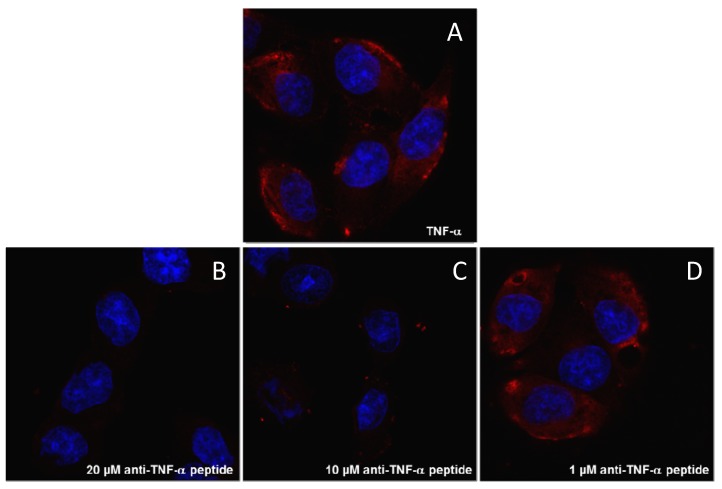
The branched anti-TNF-α peptide inhibits TNF-α binding to human melanoma cells. The red signal is due to hTNF-α binding (3 nM) to membrane receptors present on A375 cells (A). Incubation of hTNF-α with the branched anti-TNF-α peptide produces a dose-dependent decrease in the red signal (B-D). Nuclei were stained with DAPI (blue).

Human melanoma cell line A375, which expresses TNFR1 and TNFR2 [26], was used for confocal microscopy cell binding and inhibition experiments. Confocal microscopy visualizations demonstrated that the anti-TNF-α peptide inhibited binding of TNF-α to the cell surface in a concentration-dependent manner (Figure 5), confirming the inhibition results obtained by SPR (Figure 4). 

### 2.6. Branched Anti-TNF-α Peptide Dissociated Homotrimeric Form of hTNF-α

**Figure 6:**
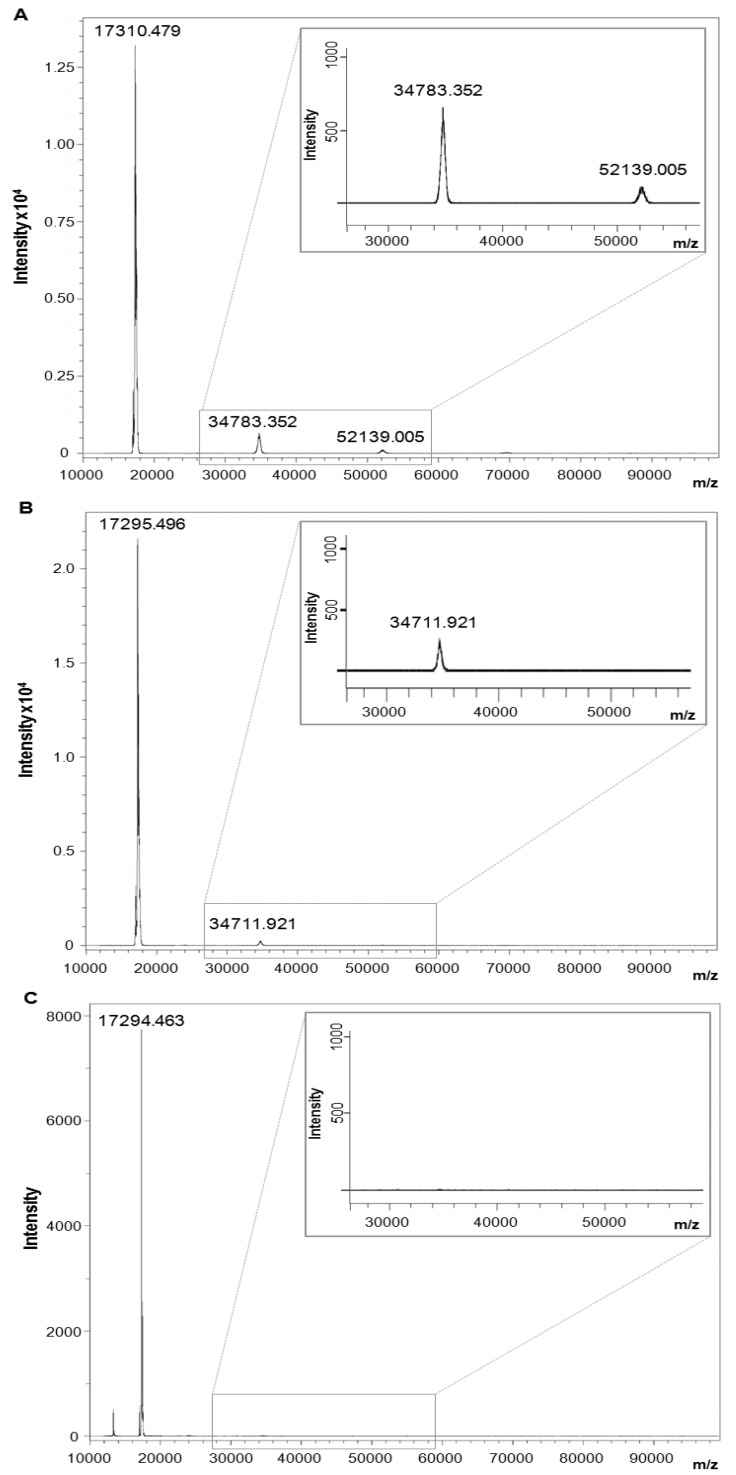
Evaluation of hTNF-α dissociation by MS. MS analysis of hTNF-α alone (**A**) or incubated with tetra-branched anti-TNF-α peptide used in a 3-fold (**B**) or 10-fold (**C**) molar excess with respect with hTNF-α. MWs are indicated over every peak. Panel A shows peaks around 17,300, 34,700 and 52,100 Da, corresponding to monomeric, homodimeric and homotrimeric forms of hTNF-α, respectively. When the concentration of anti-TNF-α peptide was increased, the peaks of trimeric and dimeric hTNF-α disappeared (B and C, respectively). The graph portion of dimeric and trimeric hTNF-α is magnified in each panel. The high intensity of monomeric TNF-α peak was the result of homo-trimer dissociation caused by laser energy generated during sample desorption. The small peak present approximately at 13,000 Da in panel C corresponds to a peptide dimer (monomer MW 6,680).

To determine whether the anti-TNF-α peptide destabilized the oligomeric form of hTNF-α, we evaluated the distribution of monomeric, dimeric and trimeric hTNF-α by MS in the presence of increasing concentrations of anti-TNF-α peptide. Figure 6A shows MS analysis of hTNF-α alone, indicating the peaks corresponding to monomeric, dimeric and trimeric forms. Figure 6B shows MS analysis of hTNF-α after incubation for 5 min with a threefold molar excess of the peptide with respect to TNF-α. The peak corresponding to the homo-trimeric form disappeared. With a tenfold molar excess of peptide, the homo-dimeric form also disappeared (Figure 6C).

## 3. Experimental Section

### 3.1. Biotinylation of Human TNF-α

100 µg hTNF-α (Prospec, Rehovot, Israel) was incubated with Biotin disulfide N-hydroxysuccinimide ester (Sigma Aldrich, St. Louis, MO, USA) at room temperature for 60 min. Unreacted biotin was removed by dialysis in PBS overnight at 4 °C.

### 3.2. Phage Display

Random 12-mer peptide phage library Ph.D.™-12 (New England Biolabs, Ipswich, MA, USA) was incubated with biotinylated hTNF-α. 10^11^ phage, diluted in PBS (137 mM NaCl, 2 mM KCl, 10 mM Na_2_HPO_4_, 2 mM KH_2_PO_4_, pH 7.4) containing 0.1% (v/v) Tween 20 (PBST) and 3% bovine albumin (BSA) and incubated with 50 nM biotinylated hTNF-α o/n at 4 °C, was added to 100 μL Dynabeads-M280 coupled with streptavidin (Invitrogen¸ Waltham, MA, USA), saturated with 5% BSA in PBST, for 15 min at room temperature. After washing 10 times with PBST, bound phages were eluted with 5 μM adalimumab for 60 min at room temperature. The amplified phages were titered and underwent two further rounds of biopanning as described above, except that the concentration of biotinylated hTNF-α was diminished (20 nM and 10 nM for the second and third rounds of panning, respectively) and washing was performed using 0.5% (v/v) Tween 20.

In alternative to the selection described above we performed the following panning using SPR technology. Biotinylated hTNF-α was immobilized to the streptavidin (SA) sensor chip surface, obtaining final 2000 Resonance Units. 10^11^ phage, diluted in HBS-EP + (10 mM Hepes, 157 mM NaCl, 2.4 mM EDTA, p20 0.05% pH 7.4), was injected over the immobilized hTNF-α at a flow rate of 10 μL/min. The elution was performed with 0.5 μM of adalimumab monoclonal antibody, and the eluted phages were collected through the Biacore T100 recovery system. Running buffer was HBS-EP +. This panning was performed with only one round of selection. For both pannings, the binding of selected clones to hTNF-α after the last round of panning was evaluated by phage ELISA in streptavidin-coated 96-well plates coated with 20 nM biotinylated hTNF-α.

### 3.3. Peptide Synthesis

Solid-phase synthesis was carried out on a MultiSynTech Syro automated multiple peptide synthesizer (Witten, Germany), employing Fmoc chemistry with 2-(1*H*-benzotriazole-1-yl)-1,1,3,3-tetramethyluronium hexafluorophosphate/N,N-diisopropylethylamine (HBTU) activation. Side chain protecting groups were trityl for His, 2,2,4,6,7-pentamethyldihydrobenzofuran-5-sulfonyl (Pbf) for Arg, *tert*-butyl ether (tBu) for Ser and Tyr, *tert*-butyl ester (OtBu) for Asp and Glu, and *tert*-butyloxycarbonyl (Boc) for Trp. Branched peptides were synthesized on Fmoc-4-Lys2-Lys Tentagel resin. For biotinylated peptides, Fmoc-Lys-(Biotin)-OH (Iris Biochem GmbH, Marktredwitz, Germany) was used as first group coupled to the solid phase and Fmoc-PEG-OH (Iris Biochem GmbH) as second. Peptides were cleaved from the resins and deprotected by treatment with trifluoroacetic acid containing water and triisopropylsilane (95:2.5:2.5). After precipitation with diethyl ether, HIHDDLLRYYGW branched peptides were purified by RP-HPLC. Final peptides purity was confirmed to be over 99% by HPLC on C18 Jupiter column (Phenomenex, 300 Å, 5 μm, 250 × 4.6 mm) using 0.1% TFA/water as eluent A and methanol as eluent B with a linear gradient from 80:20 A/B to 5:95 A/B in 30 min. Linear peptides, not purified, are >85% pure (HPLC). All peptides were characterized by UltraflexIII MALDI TOF/TOF mass spectrometry (Bruker Daltonics, Bremen, Germany).

### 3.4. Surface Plasmon Resonance (SPR).

All the experiments were performed on a Biacore T100 instrument (GE Healthcare, Milan, Italy); materials were purchased from GE Healthcare unless otherwise specified. Adalimumab was a kind gift from Galeazzi, University of Siena. Biotinylated linear and tetra-branched TNF-peptides were immobilized on a SA sensor chip. Briefly, peptides diluted in HBS-EP+ were injected for 60 s at a flow rate of 10 µL/min, obtaining 300 and 700 RUs respectively. Different concentrations of hTNF-α, from 250 nM to 2 µM in HBS-EP+, were injected for 180 s at a flow rate of 75 µL/min onto immobilized peptides. Association and dissociation kinetic rate constants (kon and koff) and the equilibrium dissociation constant Kd were calculated using BIAevaluation 3.0 software. Regeneration of the matrix was achieved with a short pulse of 10 mM NaOH. Adalimumab monoclonal antibody (1 µg/mL in HBS-EP+ for 45 s at a flow rate of 10 µL/min) was immobilized on a CM5 sensor chip previously coated with Protein A (6500 RUs). Competition experiments were carried out injecting 10 nM hTNF-α for 180 s at flow rate of 30 µL/min with various concentrations (1, 10 and 25 µM) of tetra-branched TNF-peptide. Regeneration was achieved with a 30-second pulse of glycine 10 mM pH 2.2. Human TNFR2 fused to an antibody FC fragment (TNFR Human, Prospec), diluted in HBS-EP+ at a concentration of 10 µg/mL, was injected for 120 s at a flow rate of 5 µL/min on a CM5 sensor chip previously coated with Protein A (6500 RUs). Competition experiments were carried out injecting 3 nM hTNF-α for 180 s at flow rate of 20 µL/min with various concentrations (from 1 µM to 10 µM) of tetra-branched TNF-peptide. Regeneration was achieved with a 30-second pulse of glycine 10 mM pH 2.2.

### 3.5. Cell Cultures

A375 human melanoma cells (ATCC) were grown in DMEM, supplemented with 10% fetal bovine serum, 200 mg/mL glutamine, 100 mg/mL streptomycin, and 60 mg/mL penicillin. Cells were maintained at 37 °C in 5% CO_2_.

### 3.6. Confocal Microscopy

A375 cells were plated at a density of 3 × 10^4^ per well in 24 well plates with cover glass slides. Samples were fixed through incubation with a PBS-4% paraformaldehyde (PFA) solution for 15 min, saturated for 30 min at 37 °C with PBS-1% BSA and incubated with 500 nM biotinylated hTNF-α and different concentration (20, 10 and 1 µM) of anti-TNF-α peptide for 30 min at room temperature, then with 0.5 μg/mL Streptavidin-Atto 550 (Sigma-Aldrich) for 15 min at room temperature. Nuclei were stained with DAPI. hTNF-α binding to receptors was analyzed by confocal laser microscope (Leica TCS SP5) with 364/555 nm excitation and 458/570 nm emission filters for DAPI and Atto 550, respectively. All images were processed using ImageJ software (NIH).

### 3.7. Mass Spectrometry Analysis of TNF-α Dissociation

The anti-TNF-α peptide was incubated in a 3-fold or 10-fold molar excess with respect with hTNF-α at room temperature for 5 min. The dissociation of hTNF-α was analysed by mass spectrometry using a sinapinic acid matrix (Sigma-Aldrich) and a protein standard II (mass range ~10,000–70,000 Da, Brucker Daltonics) in an ultrafleXtreme MALDI-TOF/TOF mass spectrometer (Bruker Daltonics) in linear mode.

## 4. Conclusions

TNF-α is an attractive target for the development of anti-inflammatory drugs because it is an important mediator in the pathogenesis of several inflammatory diseases, including RA, Crohn᾽s disease and ankylosing spondylitis. Very severe symptoms are already treated with anti-TNF-α recombinant antibodies or recombinant receptors, that act by sequestering the soluble TNF-α and preventing its binding to receptors, or activating defense mechanisms such as ADCC and CDC [27]. Five anti-TNF-α agents have been successfully introduced into clinical practice for chronic inflammatory diseases [14] but they have some major side effects [28,29,30,31]. Since they are also very expensive it is important to identify new anti-TNF-α agents to enlarge the panel of possible therapeutic agents for inflammatory diseases.

In this study we identified a new anti-TNF-α molecule using a peptide combinatorial library. Differently to other authors which used the same technology for the recovery of anti-TNF-α peptides [32], we used a competitive elution system to directly rescue specific peptide ligands, a procedure successfully tested by us in different applications [21,33]. Here the human monoclonal antibody Adalimumab (Humira^®^) was used for competitive elution of phage peptides. Adalimumab is also derived from phage library selection and subsequent optimization. It has been marketed since 2002 for the treatment of severe symptoms of RA and other inflammatory pathologies. It is a high affinity ligand of soluble TNF-α and as such competed strongly with phage peptides possibly bound to TNF-α. Competitive detachment of phage peptides during the panning phase made it possible to select a peptide that bound TNF-α in a protein site included in the antigen epitope recognized by adalimumab, thus obtaining a sort of mimotope of the adalimumab binding site. Interestingly, although we used a library consisting of billions of different clones, we found only one peptide sequence that bound TNF-α, even using two different selection strategies: one incubating phages with the soluble target in a tube, the other flowing the phage library directly on a Biacore sensor chip where the target was immobilized.

The peptide identified by phage selection competed with adalimumab and significantly also with natural receptors of TNF-α. This inhibition was demonstrated at molecular level by Biacore (Figure 4) and directly on viable cells expressing TNF-α receptors (Figure 5). The latter result suggests that the peptide may inhibit the onset of inflammation-boosting reactions triggered by TNF-α on cells.

MS experiments demonstrated that the peptide dissociated the oligomeric form of TNF-α, suggesting a mechanism of action that entails the disruption of receptor binding. However, we cannot exclude the possibility that sequestration of the active trimeric form contributes to an increase in inhibitory activity.

The peptide used in these experiments was synthesized in the tetra-branched form, which is considered particularly suitable for *in vivo* use by virtue of its stability to peptidase and proteases of fluids and organs. The prolonged half-life of branched peptides has been extensively demonstrated by us with several branched peptides used in different applications [20,21,22,23,24,25]. The production of peptides in branched form is a well-established procedure that enables production of very uniform batches of peptides at costs compatible with industrial application. Synthesis in branched form can be considered a first optimization phase for further characterizations in preclinical tests. This work is part of the lead identification and optimization stages necessary for preclinical characterization aimed at development of a new drug for human use.
